# Oxidative Stress Down-Regulates MiR-20b-5p, MiR-106a-5p and E2F1 Expression to Suppress the G1/S Transition of the Cell Cycle in Multipotent Stromal Cells

**DOI:** 10.7150/ijms.38832

**Published:** 2020-02-04

**Authors:** Lihui Tai, Chiu-Jung Huang, Kong Bung Choo, Soon Keng Cheong, Tunku Kamarul

**Affiliations:** 1 Centre for Stem Cell Research, Faculty of Medicine and Health Sciences, Universiti Tunku Abdul Rahman, Selangor, Malaysia; 2 Postgraduate Program, Faculty of Medicine and Health Sciences, Universiti Tunku Abdul Rahman, Selangor, Malaysia;; 3 Department of Animal Science & Graduate Institute of Biotechnology, Chinese Culture University, Taipei, Taiwan;; 4 Department of Preclinical Sciences, Faculty of Medicine and Health Sciences, Universiti Tunku Abdul Rahman, Selangor, Malaysia; 5 Dean's Office, Faculty of Medicine and Health Sciences, Universiti Tunku Abdul Rahman, Selangor, Malaysia;; 6 Tissue Engineering Group, National Orthopedic Centre of Excellence for Research and Learning & Department of Orthopedic Surgery, Faculty of Medicine, Universiti Malaya, Kuala Lumpur, Malaysia

**Keywords:** oxidative stress, miR-20b-5p & miR-106a-5p, miR-17 family, p21/CDK/E2F1 pathway, E2F1, G1/S transition of cell cycle

## Abstract

Oxidative stress has been linked to senescence and tumorigenesis via modulation of the cell cycle. Using a hydrogen peroxide (H_2_O_2_)-induced oxidative stress-induced premature senescence (OSIPS) model previously reported by our group, this study aimed to investigate the effects of oxidative stress on microRNA (miRNA) expression in relation to the G1-to-S-phase (G1/S) transition of the cell cycle and cell proliferation. On global miRNA analysis of the OSIPS cells, twelve significantly up- or down-regulated miRNAs were identified, the target genes of which are frequently associated with cancers. Four down-regulated miR-17 family miRNAs are predicted to target key pro- and anti-proliferative proteins of the p21/cyclin D-dependent kinase (CDK)/E2F1 pathway to modulate G1/S transition. Two miR-17 miRNAs, miR-20-5p and miR-106-5p, were confirmed to be rapidly and stably down-regulated under oxidative stress. While H_2_O_2_ treatment hampered G1/S transition and suppressed DNA synthesis, miR-20b-5p/miR-106a-5p over-expression rescued cells from growth arrest in promoting G1/S transition and DNA synthesis*.* Direct miR-20b-5p/miR-106a-5p regulation of p21, CCND1 and E2F1 was demonstrated by an inverse expression relationship in miRNA mimic-transfected cells. However, under oxidative stress, E2F1 expression was down-regulated, consistent with hampered G1/S transition and suppressed DNA synthesis and cell proliferation. To explain the observed E2F1 down-regulation under oxidative stress, a scheme is proposed which includes miR-20b-5p/miR-106a-5p-dependent regulation, miRNA-E2F1 autoregulatory feedback and E2F1 response to repair oxidative stress-induced DNA damages. The oxidative stress-modulated expression of miR-17 miRNAs and E2F1 may be used to develop strategies to retard or reverse MSC senescence in culture, or senescence in general.

## Introduction

Oxidative stress is a physiological process induced by reactive oxygen (ROS) and/or nitrogen species 1,2. Highly reactive ROS include superoxide anion (O_2_^-^) and hydrogen peroxide (H_2_O_2_), which may cause damages to macromolecules and consequently the associated cellular and metabolic processes. Hence, oxidative stress is associated with aging and diseases, including cancer 2-6. Cells are, fortunately, equipped with antioxidant defense mechanisms 7,8.

Multipotent stromal cells (MSC) are able to self-renew and exhibit multilineage differentiation ability, thus, presenting great potentials in therapeutic applications in regenerative medicine and cell therapy 9,10. However, proliferating MSC ages rapidly in culture, hampering therapeutic potentials 10,11. The mechanism of such debilitation is not well understood. We have previously reported the establishment of oxidative stress-induced premature senescence (OSIPS) in Wharton's Jelly (WJ)-MSC by treatment with H_2_O_2_ 12. H_2_O_2_ treatment of cells causes accumulation of ROS to provoke oxidative stress, which, in turn, induces DNA single- and double-strand breaks and telomere losses to accelerate senescence 13,14. It has been also established that the onset of senescence involves hampered self-renewal properties through disruption of the G1-to-S-phase (G1/S) transition of the cell cycle 15,16.

One of the important regulators of gene expression is the microRNA (miRNA). MiRNAs are small single-stranded non-coding RNAs that regulate expression of target genes via degradation or translational repression of the targeted transcripts. Hence, miRNAs control a broad range of cellular functions in stem cells, including self-renewal, proliferation and regulation of G1/S transition of the cell cycle 17-19. Several miRNAs have previously been reported to be differentially expressed in senescence cells 19-23. MiRNAs have also been reported to regulate oxidative stress-induced cellular senescence (reviewed in 24). However, details on pathways that depict interactions between miRNAs and cell-cycle factors in senescence cells remain to be fully elucidated.

The G1/S transition of the cell cycle is closely associated with senescence. Using the OSIPS MSC model, this work focused on further elucidating the biochemical and cellular effects of oxidative stress on miRNA expression in regulating the G1/S phase of the cell cycle. Differentially expressed miRNAs and the target transcripts in the H_2_O_2_-induced oxidative-stressed MSC were identified, and selected miRNA-targeted genes in the p21/cyclin D-dependent kinase (CDK)/E2F1 pathway were verified by functional studies. The data show that down-regulated expression of miR-20b-5p and miR-106a-5p, both members of the miR-17 family, contribute to regulation of G1/S-phase transition of the cell cycle via promoting p21 expression and suppressing E2F1 in oxidative-stressed multipotent stromal cells.

## Materials and Methods

### Cell culture of Wharton's Jelly-derived MSC (WJ-MSC)

Human multipotent stromal cell lines (previously called mesenchymal stem cells in 12) at passage 3 to 5, WJ0706, WJ0619 and WJ2000, derived from Wharton's Jelly (WJ) of the umbilical cord, were obtained with compliments from Cryocord Sdn Bhd, Selangor, Malaysia (http://www.cryocord.com.my), and has previously been described 12. The cell lines were isolated and the MSC phenotype was confirmed at Cryocord as previously described 25. WJ-MSC was cultured in complete Dulbecco's Modified Eagle Medium composed of nutrient mixture F12 (DMEM/F12) (Gibco, NY, USA) supplemented with 10% fetal bovine serum (Gibco) and 1% penicillin/streptomycin. The medium was changed every 3 days and cells were re-plated at a density of 5,000 cells/cm^2^ upon reaching 80% confluency. All cells were cultured at 37 ^o^C in 5% carbon dioxide.

### Hydrogen peroxide (H_2_O_2_) treatment

H_2_O_2_ treatments were performed on confluent cells (3.75 x 10^5^ cells per T75 flask) as previously described 12. In brief, cells at passage 6 to 8 were incubated in complete DMEM/F12 medium containing 200 µM H_2_O_2_ for 2 h; cells were washed and further cultured in fresh complete DMEM/F12 medium without H_2_O_2_ for 24 h for recovery before being harvested for subsequent assays. In the time-course experiments, the cells were treated with 200 µM H_2_O_2_ for different durations, followed by cell recovery for 24 h before RNA preparation. In the miRNA stability test, the H_2_O_2_-treated cells were cultured in complete medium for different time points before being harvested for RNA preparation.

### miRNA microarray analysis

For miRNA profiling analysis, the Gen. 7 version of miRCURY LNA™ microRNA Arrays (Exiqon, Vedbaek, Denmark), which included validated and T_m_-normalized LNA™-based capture probes, were used. The arrays included 1,896 of the 1,921 annotated human miRNAs in the miRBase 18.0. Included in the arrays were also 25 proprietary miRPlus™ human miRNAs not yet annotated in miRBase. The arrays were done in four replicate spots. The quality of the total RNA was verified by an Agilent 2100 Bioanalyzer profile. Only total RNA preparations with RNA Integrity (RIN) values > 7 were used in the array. Total RNA (450 ng) from both the experimental samples and the reference was labeled with Hy3™ or Hy5™ fluorescent label, respectively, using the miRCURY LNA™ microRNA Hi-Power Labeling Kit, Hy3™/Hy5™ (Exiqon) following established procedure of the manufacturer. The Hy3™-labeled experimental and a Hy5™-labeled reference RNA samples were mixed pair-wise and hybridized to the miRCURY LNA™ microRNA Array using a Tecan HS4800™ hybridization station (Tecan, Grödig, Austria). The array slides were scanned using the Agilent G2565BA Microarray Scanner System (Agilent Technologies Inc., CA) and image analysis was carried out using the ImaGene® 9 microRNA Array Analysis Software (Exiqon). The quantified signals were background-corrected (Normexp with offset value 10, see 26 and normalized using the global LOcally WEighted Scatterplot Smoothing (LOWESS) regression algorithm. For the present data set, a total of 1,469 probes were discarded by this filtering procedure, leaving 564 probes detectable above the background threshold for each sample. Unsupervised hierarchical clustering was performed using the complete-linkage method and the Euclidean distance measure.

### RNA isolation, reverse transcription and quantitative RT-PCR

Total RNA was isolated from WJ-MSC using RNeasy Plus Mini Kit (Qiagen, CA, USA). For miRNA expression analysis, miRNA polyadenylation was first performed using 1 μg total RNA, followed by first-strand cDNA synthesis using the NCode™ miRNA first-strand cDNA synthesis and qRT-PCR kit (Invitrogen, CA, USA). Ten-fold diluted cDNA was used for quantitative RT-PCR using a SYBR select master mix kit (Applied Biosystems, TX, USA) in a Rotor-Gene Q (Qiagen) thermal cycler. The primers used for qRT-PCR analysis are shown in Supplementary Table S1. PCR was carried out under the following conditions: 50 °C for 2 min, 95 °C for 10 min followed by 40 cycles of 95 °C for 15 s and 60 °C for 1 min. Data was analyzed by using the comparative ΔΔC_t_ method, and miRNA expression was normalized to the expression level of U6. All miRNA quantification experiments were performed in triplicates, and the data presented were derived from three independent experiments.

### miRNA target prediction, ontology and KEGG pathway analysis

miRNA target transcripts were predicted using the web-based algorithms miRBase (www.mirbase.org), TargetScan Release 7.1 (http://www.targetscan.org/), DIANA-microT-CDs v5.0 (http://diana.imis.athena-innovation.gr) and miRTarBase (www.mirtarbase.mbc.nctu.edu.tw). Ontology and KEGG pathway analysis were performed by using DAVID Bioinformatics Resources v6.8 27,28.

### Protein extraction and western blotting analysis

Total protein lysates were prepared using the RIPA buffer (50 mM Tris•HCl pH 7.6, 150 mM NaCl, 1% NP-40, 0.5% sodium deoxycholate, protease inhibitor cocktail, 0.1% SDS) (Nacalai Tesque, Kyoto, Japan). Western blot analysis was performed as previously described 29 using the following rabbit primary antibodies: p21 (Cell Signaling, MA, USA), E2F1 (Abcam), CCND1 (Abcam) or GAPDH (Cell Signaling). The relative optical density values of the proteins bands were quantified by comparing the H_2_O_2_-treated cells relative to untreated cells, or miRNA mimic-transfected cells relative to the negative mimic control cells, after normalization to the GAPDH loading control using Image J software version 1.49v (National Institutes of Health, MD, USA).

### Luciferase reporter assay

PCR products of full-length or segments of 3'-untranslated region (3'UTR) of the target genes harboring the predicted miR-20b-5p or miR-106a-5p binding sites were cloned into the dual luciferase (*Firefly/Renilla*) reporter vector pmirGLO using the restriction enzyme combinations SacI/XbaI or SalI/XhoI^30^. The colorectal cancer cell line HCT-15 was co-transfected in 24-well plates using 1µl Lipofectamine 2000 (Invitrogen) with 200 ng empty pmirGLO or the pmirGLO/3'-UTR construct, and 10nM miRNA mimic or a validated nonspecific negative control miRNA mimic (Ambion, CA, USA) and the subsequent luciferase assay was performed as described 29.

### Transfection of miRNA mimic and BrdU assays

To test the effects of miRNA over-expression, WJ0706 cells were transfected with 25 nM or 50 nM of miRNA mimics, or a validated nonspecific negative control miRNA mimic (Ambion) in 24-well plates using 1µl Lipofectamine2000 (Invitrogen) according to the manufacturers' protocols. Twenty-four hours post transfection, fresh medium was changed and the cells were harvested 48 h post-transfection for BrdU assays (Cell Signaling Technology) in triplicates in three independent biological experiments as described 29.

### Cell cycle analysis

Twenty-four hours post-H_2_O_2_ treatment and forty-eight hours post-miRNA transfection, the cells were harvested and were fixed overnight with 70% cold ethanol at -20 °C. The cells were then treated with 1mg/mL RNase A and stained with 10µg/mL propidium iodide in the dark for 30 min at 37 ^o^C, followed by analysis on a FACS Canto-II analyzer (BD Biosciences, CA, USA). A total of 100,000 events were recorded for each sample. The experiments were done three times independently before quantification and statistical analysis.

### Statistical analysis

Statistical analysis was performed in Microsoft Excel 2010 using the statistical formula to obtain the mean values, standard deviation and variance. Real-time RT-PCR results were reported as average of log (fold change) ± standard error. All data were analyzed by Student's *t* test using the negative controls as reference. Statistical significance was accepted at *p*<0.05.

## Results

### Global expression analysis identifies deregulated miRNAs associated with signaling processes, cell cycle regulation and tumorigenesis under oxidative stress

**Figure 1:**
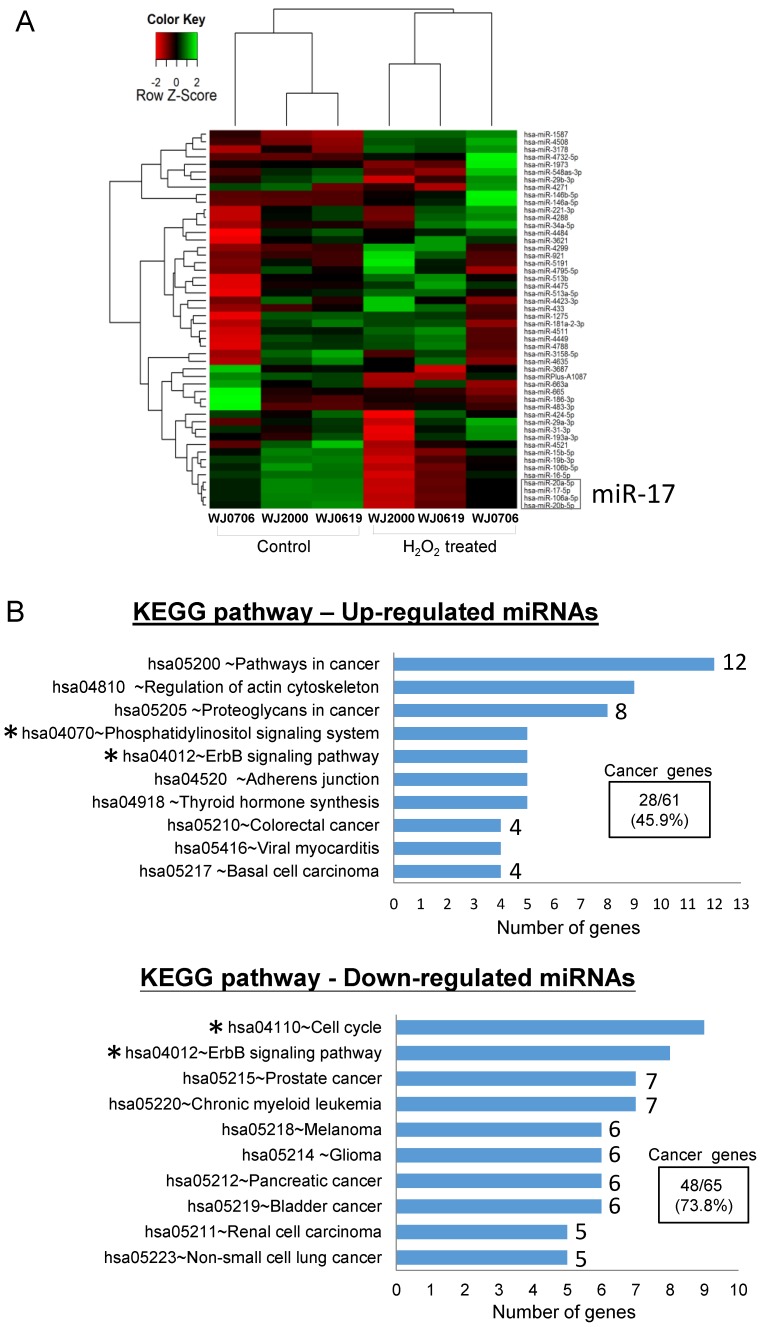
Differentially expressed miRNAs in oxidative-stressed multipotent stromal cells.** (A)** Heatmap and unsupervised hierarchical clustering of the top fifty differentially expressed miRNAs in three H_2_O_2_-treated WJ-MSC lines WJ0706, WJ2000 and WJ0619 (see Supplementary Table S2 for a full list). Color code for relative miRNA expression levels: red and green represent expression levels below and above the reference channel, respectively. Normalized log_2_(fold change) ratio values were used for the analysis. The down-regulated miR-17 family miRNAs that were further investigated in this work are boxed (see also Table 1). **(B)** Top 10 KEGG pathways of the differentially expressed miRNAs. The number of predicted genes in each cancer-associated pathway is shown on the right end of the bar, and the percentage of the total number of the predicted cancer genes is shown in the box. Asterisks indicate pathways that are associated with signaling or the cell cycle (see also Supplementary Table S3).

To identify oxidative stress-associated miRNAs in the OSIPS cells, global miRNA expression profiles of three WJ-MSC lines, WJ0706, WJ0619 and WJ2000, and the respective H_2_O_2_-treated cells, were established by microarray analysis. Only twenty-eight up-regulated and twenty-two down-regulated miRNAs were identified in the H_2_O_2_-treated cells relative to the untreated cells (Supplementary Table S2). A heatmap was constructed using the fifty deregulated miRNAs for unsupervised hierarchical clustering analysis: the data were clustered into the control (untreated) and H_2_O_2_-treated groups as anticipated (Figure 1A). Out of the fifty deregulated miRNAs, only seven up-regulated miRNAs answered to the criteria of log_2_ (fold change)>1.0 and *p*<0.05, and five down-regulated miRNAs fulfilled the criteria of log_2_(fold change) <-1.0 and *p*<0.05 (Table 1); the twelve miRNAs were further subjected to gene ontology and KEGG pathway analyses. Amongst the top 10 affected biological and molecular processes (Supplementary Table S3), most notable are 258 genes expressed in processes associated with gene regulation (gene count n=85, 32.9%), kinase activity and signaling (n=42, 16.3%) and cell cycle regulation (n=5, 1.9%) (Table 2). In particular, the targeted processes of post-transcriptional gene silencing by RNA (GO:0035194) and miRNA-mediated inhibition of translation (GO:0035278) are consistent with the post-transcriptional regulation properties of miRNAs. KEGG analysis also predicts involvement of signaling pathways (hsa04012 & hsa04070, n=13) and the cell cycle process (hsa04110) (Figure 1B, asterisked) in both the up- and down-regulated miRNA groups. It is noteworthy that twenty-eight (45.9%) of the sixty-one predicted genes in the KEGG pathways of the up-regulated miRNAs, and forty-eight (73.8%) of the sixty-five down-regulated miRNAs are associated with pathogenesis pathways of various cancers (Figure 1B).

**Figure 2:**
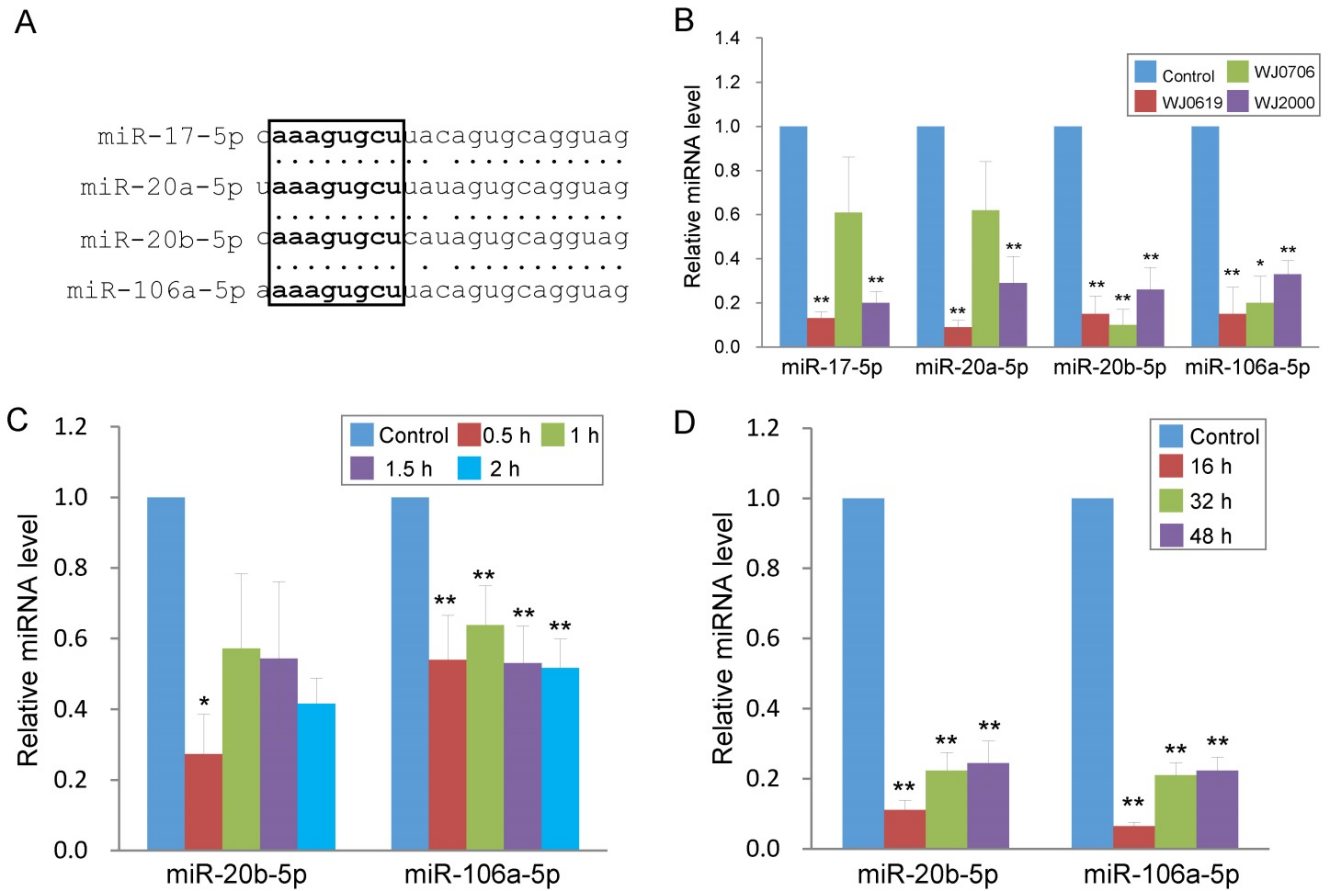
Oxidative stress induces long-term down-regulation of miR-20b-5p and miR-106a-5p. **(A)** Sequence alignment of the miR-17 family miRNAs. The seed sequence is in bold letters and is boxed. **(B)** Validation of down-regulated expression of the miR-17 family miRNAs in three H_2_O_2_-treated MSC lines by real-time RT-PCR. The control was cells untreated with H_2_O_2_. **(C, D)** Rapid down-regulation of miR-20b-5p and miR-106a-5p in MSC on H_2_O_2_ treatment. MiRNA expression was assayed in WJ0706 cells treated with 200 µM H_2_O_2_ for 0.5 to 2 h **(C)**, or after the 2-h H_2_O_2_ treatment, the washed cells were further cultured for an extended period of time in fresh medium without H_2_O_2_ **(D)** before real-time RT-PCR analysis. *p<0.05 and **p<0.01 were relative to the untreated control samples.

In the up-regulated miRNA group (Table 1), miR-146a-5p and miR-146b-5p have previously been shown to be deregulated in expression in late-passage fibroblast cells 31. The remaining five up-regulated miRNAs were first reported in deep sequencing of small RNA transcriptomes of several types of cancer 32-36. In the down-regulated group, miR-16-5p is a tumor suppressor miRNA reportedly involved in regulating the cell cycle and apoptosis 37, consistent with the gene ontology data above (Figure 1B & Table 2). The remaining down-regulated miRNAs all belong to the miR-17 family and are highly homologous in the sequences, and share the same seed sequence (Figure 2A) 38. Down-regulated expression of the four miR-17 family members was confirmed in real-time RT-PCR analysis in the three WJ-MSC cell lines after H_2_O_2_ treatment (Figure 2B). Of the four miR-17 miRNAs, the chromosome 13-associated miR-17-5p and miR-20a-5p have previously been studied in relation to differentiation, tumorigenesis and the G1-to-S-phase transition of the cell cycle 30, 39-40. However, the chromosome X-linked miR-20b-5p and miR-106a-5p have not previously been investigated, and were the focus of the present work in relation to oxidative stress-induced deregulation of the G1/S-phase transition.

To first investigate the expression kinetics of miR-20b-5p and miR-106a-5p under oxidative stress, WJ0706 cells were treated with H_2_O_2_ for various periods of time. Data showed that H_2_O_2_ treatment for 0.5 h was sufficient to rapidly down-regulate expression of both miRNAs, and the suppressed miRNA levels were maintained for up to 2 h (Figure 2C). The data, thus, established a direct and rapid responsiveness of miR-20b-5p/miR-106a-5p to H_2_O_2_-induced oxidative stress. It was further shown that after 2 h H_2_O_2_ treatment, the suppressed miRNA expression levels were maintained for up to 48 h in the cell recovery period in fresh medium without H_2_O_2_ (Figure 2D), indicating irreversible long-term miRNA down-regulation in the oxidative-stressed cells.

### Oxidative stress suppresses while miR-20b-5p and miR-106a-5p promote cell proliferation, G1/S transition and DNA synthesis

**Figure 3:**
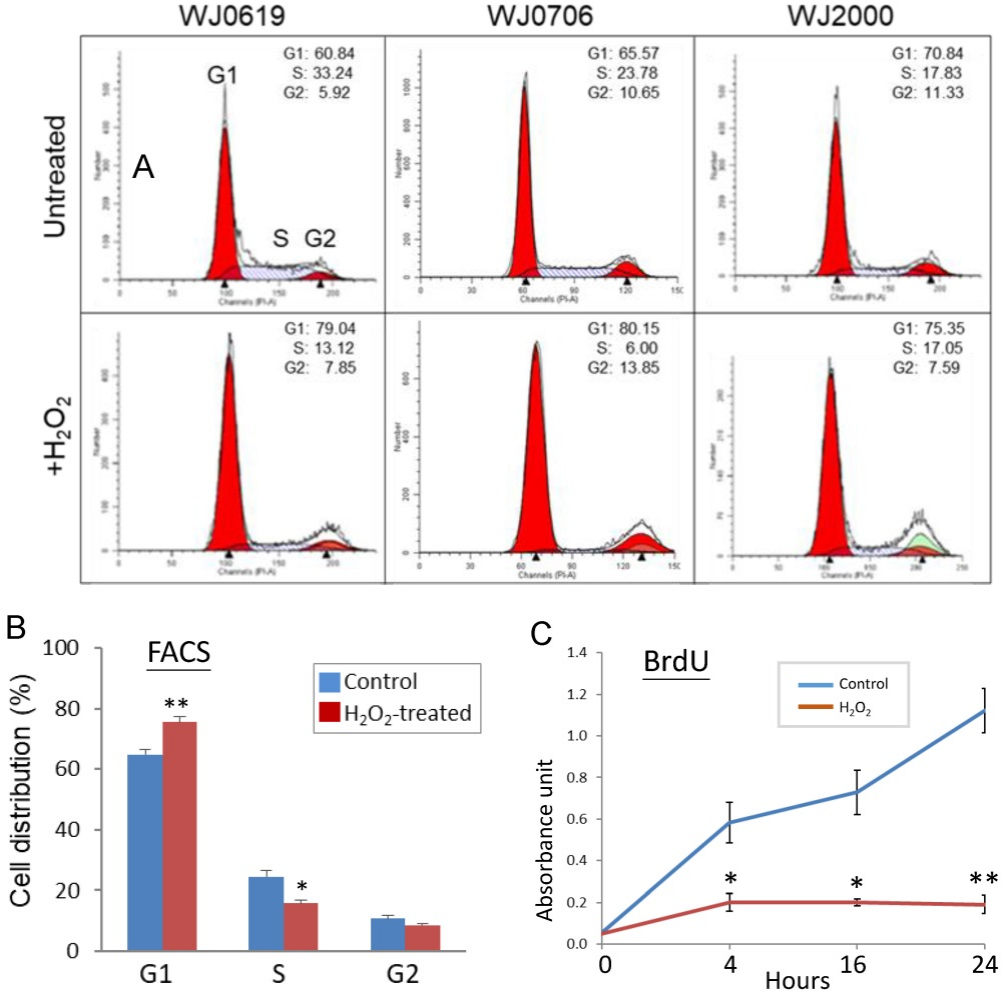
Oxidative stress suppresses G1-to-S-phase transition of the cell cycle and DNA synthesis.** (A, B)** Effects of H_2_O_2_ treatment on G1/S transition. In the flow cytometry analysis, a representative dataset of the of the three cell lines tested after treatment with 200 µM H_2_O_2_ for 2 h is shown in **(A)**; the mean values of three independent experiments and combining data of the three cell lines is shown in **(B)**. **(C)** DNA synthesis rate of H_2_O_2_-treated cells determined by BrdU assays at different time points. All the data shown are the means of three independent experiments. *p<0.05 and **p<0.01 were relative to the untreated cells.

In stem cells, the G1/S transition of the cell cycle is accelerated 15, 41-42 while it is retarded in senescence cells 43-45. The effects of H_2_O_2_-induced oxidative stress on the cell cycle were evaluated in flow cytometric (FACS) analysis. Representative FACS profiles of the three cell lines analyzed are shown in Figure 3A; combined data of three independent experiments of the three cell lines tested are shown in Figure 3B. The results showed that the fractions of G1-phase cells significantly increased on H_2_O_2_ treatment, with concurrent declines of the S1 and G2 cell populations, indicating hampered G1/S transition 46. To obtain supporting evidence, DNA synthesis rates of H_2_O_2_-treated cells were determined in BrdU assays (Figure 3C). The data, indeed, showed that on H_2_O_2_ treatment, DNA synthesis rates were significantly suppressed, supporting that the G1-phase cells were blocked, or delayed, from entry into the S phase.

**Figure 4:**
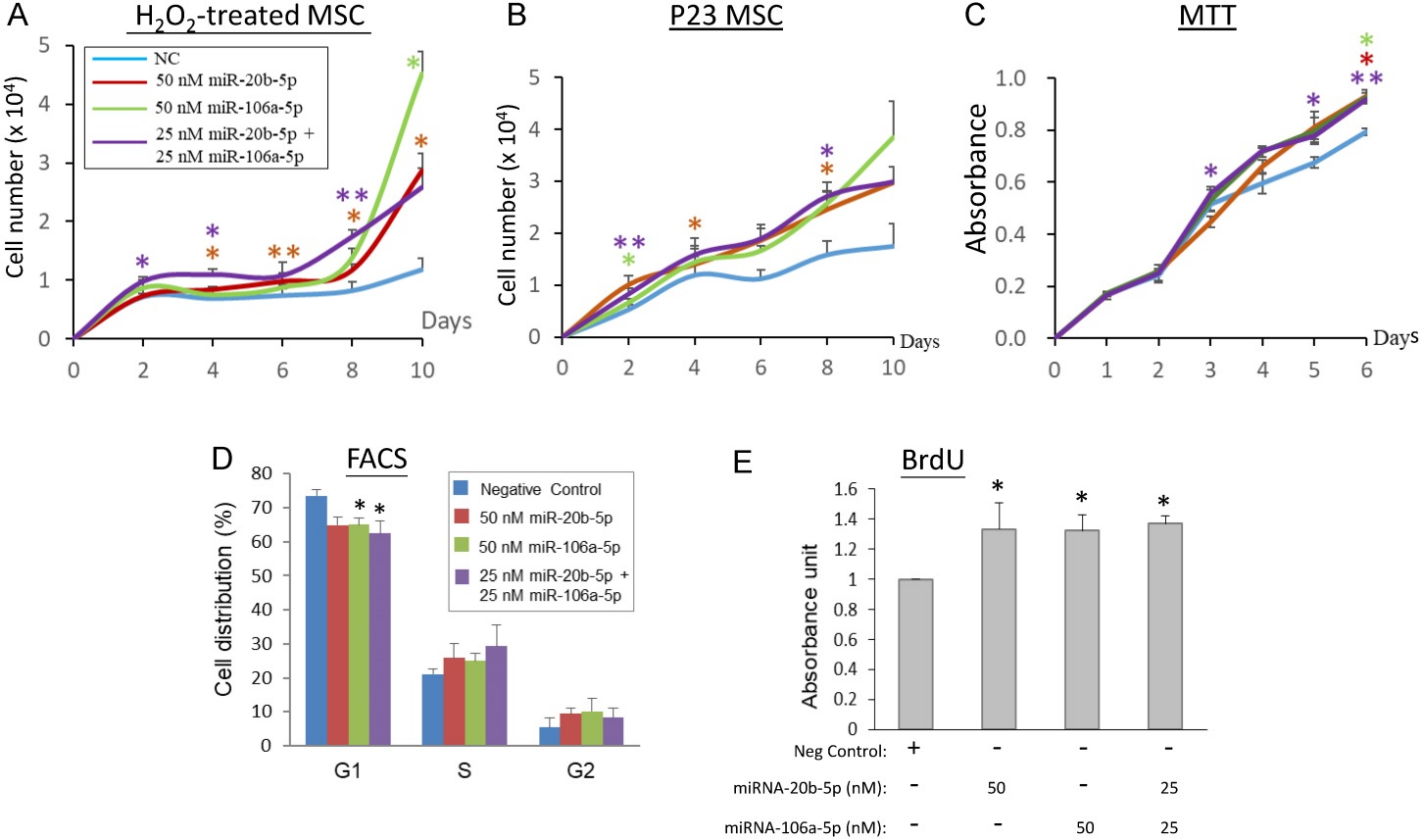
MiR-20b-5p and miR-106a-5p promote cell proliferation, G1/S transition and DNA synthesis.** (A, B)** Effects of over-expression of miRNA mimics on cell growth in H_2_O_2_-treated **(A)** or late-passage (P23) replicative senescence (RS) WJ0706 cells **(B)**. **(A)** Transfection of miRNA mimics was performed for 24 h**,** followed by 200 µM H_2_O_2_ treatment for 2 h before the cells were washed and cultured in fresh medium without H_2_O_2_. Cells were counted on the specified days post treatment. **(B)** Growth curve of miRNA-transfected P23 RS cells without H_2_O_2_ treatment.** (C - E)** Effects of over-expression of the miRNA mimics in WJ0706 cells on cell viability, determined by MTT analysis **(C)**, distribution of G1-, S- and G2-phase cells, determined by flow cytometry (FACS) analysis **(D)**, and DNA synthesis rate, determined by BrdU analysis **(E)**. In **(A-C)**, only the top halves of the error bars are shown; asterisks shown in different colors indicate statistical significance matching the different experimental curves of the same colors. The color codes for the miRNA transfection shown in (A) also apply to (B) & (C). The blue curves are the no-miRNA transfection negative controls. Data presented were from three independent experiments. *p<0.05 and **p<0.01 were relative to the nonspecific negative control (NC) mimic.

To elucidate the association between the miR-20b-5p and miR-106a-5p and cell proliferation, transient over-expression of the miRNA mimics in H_2_O_2_-treated and late replicative senescence (RS) WJ0706 cells was performed, followed by cell counts in cell growth analysis. Since the two miRNAs shares an identical seed sequences and a high degree of sequence homology (18/21) (Figure 2A), the interchangeability of the two miRNAs was also tested. On 2 h H_2_O_2_ treatment, followed by further culturing in H_2_O_2_-free medium, the negative miRNA mimic-transfected control cells entered growth arrest on day 2 and up to day 10. On the other hand, growth arrest of the miR-20b-5p and/or miR-106a-5p mimic-transfected cells ended when the cells entered the log phase of growth on day 6 to day 8 (Figure 4A). The growth delay was probably due to delays for the exogenous miRNAs to exert their gene regulatory and the subsequent biochemical actions. The late-passage RS cells did not seem to have a clear growth-arrest phase as shown in the early-passage cells on H_2_O_2_ treatment; however, the miRNA mimic-transfected RS cells grew more rapidly than the negative miRNA mimic-transfected control cells (Figure 4B). Furthermore, the miR-20b-5p and miR-106a-5p mimics, when used individually at 50 nM, or in combination at a combined final concentration of 50 nM, displayed similar growth patterns. The results indicate that the two miRNAs are interchangeable in exerting biological functions, echoing our previous hypothesis that miRNA redundancy provides a fail-proof scheme in miRNA regulation of crucial cellular events 47.

The biological role of miR-20b-5p and miR-106a-5p in modulating cell proliferation, the cell cycle and DNA synthesis was next investigated in MTT, flow cytometry and BrdU assays*,* respectively (Figures 4C-4E). In MTT analysis, WJ0706 cells not under oxidative stress maintained a steady growth rate both in the negative miRNA mimic-transfected control cells and in the miRNA mimic-transfected cells; however, miR-20b-5p and/or miR-106a-5p over-expression consistently resulted in higher numbers of viable cells and, thus, higher cell proliferation rates (Figure 4C). Interchangeability of the two miRNAs was again observed. Likewise, flow cytometric analysis of the miRNA mimic-transfected cells showed that either one of the miRNA mimics resulted in a significant decrease in the G1-phase cell population with concurrent increases in the S- and G2-phase cells when compared with transfection of a negative control miRNA mimic; furthermore, a significant G1 cell reduction was observed on the combined used of both the miRNA mimics (Figure 4D). The data support that miR-20b-5p and miR-106a-5p play a role in modulating the G1/S transition. The finding was further supported by data of BrdU analysis, which showed significantly enhanced DNA synthesis rates on single or co-transfection of the two miRNAs (Figure 4E).

Taken together, evidences presented in this (Figures 3 & 4) and the preceding section (Figure 2) indicate that H_2_O_2_-induced oxidative stress leads to down-regulated expression of miR-20b-5p and miR-106a-5p, and suppresses G1/S transition and DNA synthesis. On the other hand, the miR-20b-5p and miR-106a-5p over-expression enhances cell growth and proliferation, the G1/S transition and DNA synthesis. Since miRNAs are negative regulators, the H_2_O_2_ and miRNA data are consistent.

### miR-20b-5p/miR-106a-5p and oxidative stress modulate the p21/CDK/E2F pathway

**Figure 5:**
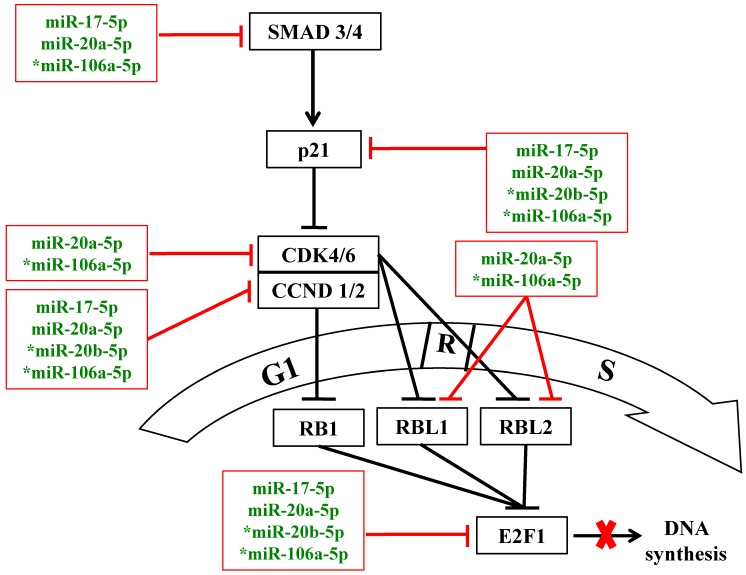
MiR-17 family miRNAs are predicted to target the p21/CDK/E2F1 pathway to modulate the G1/S transition of the cell cycle under oxidative stress. Blunted red lines indicate bioinformatics-predicted negative regulation by the miR-17 family miRNAs; asterisks indicate miRNAs that were further analyzed in this study. R: the restricted entry point of the cell cycle. The thick red cross denotes block in DNA synthesis.

In normal cells, enhanced cell proliferation may be due to more rapid G1/S-phase transition via concerted regulation of pro- and anti-proliferative factors of the p21/CDK/E2F pathway 11,17. Database interrogation and bioinformatics analysis have predicted that the miR-20a-5p and miR-106a-5p target multiple components of the p21/CDK/E2F1 pathway, which modulates the G1/S transition of the cell cycle (Figure 5), as has been forecast by KEGG pathway analysis above (Figure 1B & Table 2). In this work, further functional analysis was done on the p21 protein, the downstream cyclin D1 and D2 (CCND1/2) and E2F1.

**Figure 6:**
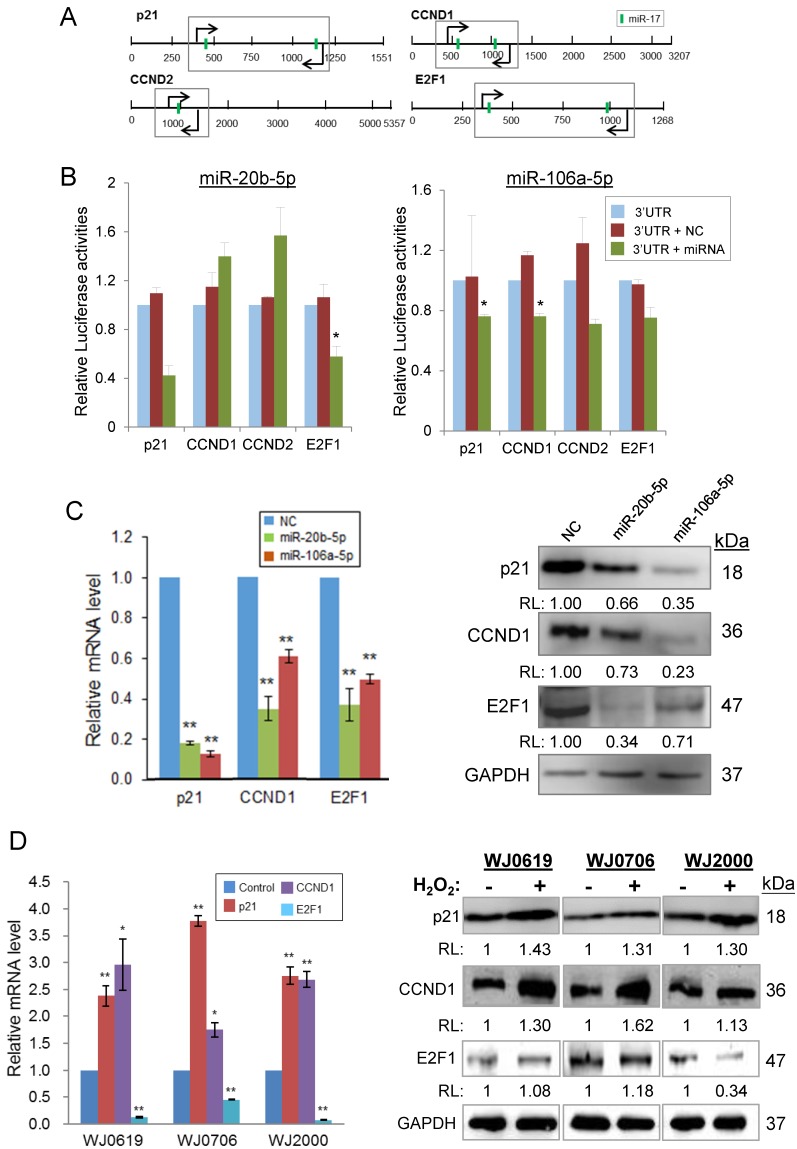
MiR-20b-5p and miR-106a-5p and oxidative stress regulate specific genes of the p21/CDK/E2F pathway.** (A)** 3'-UTR of the transcripts of genes of the p21 pathway; the predicted miR-20b-5p/miR-106a-5p target sites (vertical green bar) are shown. Bent arrows indicate the position of the PCR primers used in the amplification of the 3'UTR segments (boxed) cloned into the pmirGLO vector 30. **(B)** Validation of targeting of miR-20b-5p/miR-106a-5p by luciferase assays. HCT-15 cells were co-transfected with pmirGLO/3'UTR or the pmirGLO blank vector, and a miR-20b-5p or miR-106a-5p mimic, or a negative control mimic. *p<0.05 and **p<0.01 were relative to the data of pmirGLO/3'UTR without transfected miRNA or negative control (NC) mimic. **(C)** Inverse relationship between expression levels of miR-20b-5p/miR-106a-5p and the target transcripts and proteins in miRNA mimic-transfected cells, determined by qRT-PCR (left panel) and western blot analysis (right panel). **(D)** Effects of oxidative stress on the miRNA target transcript and protein levels in three MSC cell lines. The total RNA (left panel) and protein samples (right panel) were prepared from H_2_O_2_-treated (2 h) or -untreated cells, which were further cultured for 24 h post H_2_O_2_ treatment before being harvested for further analysis. *p<0.05 and **p<0.01 were relative to the untreated or the negative control (NC) samples. In the western blots in **(C)** and **(D)**: RL, relative levels compared to the negative control (C) or untreated control (D) samples.

MiR-20b-5p/miR-106a-5p targeting of transcripts of p21, CCND1, CCND2 and E2F1 was first verified by luciferase assays (Figures 6A & 6B). The putative miRNA target sites, which are common for both miRNAs (Figure 6A), was cloned into the dual luciferase (*Firefly/Renilla*) reporter vector pmirGLO. In the presence of a miR-20b-5p mimic, the p21 and E2F1, but not CCDN1/2, constructs showed reduced luciferase activities, whereas the miR-106a-5p mimic down-regulated luciferase expression of all four proteins (Figure 6B), signifying miRNA targeting of the transcripts. MiRNA targeting was further demonstrated by an inverse relationship in the miRNA levels and levels of the targeted transcripts and proteins in the miRNA mimic-transfected cells. Over-expression of miR-20b-5p or miR-106a-5p significantly down-regulated the p21, CCND1 and E2F1 mRNA levels (Figure 6C, left panel). Likewise, the proteins levels were also down-regulated (Figure 6C, right panel) supporting miRNA targeting.

Under H_2_O_2_-induced oxidative stress, results showed elevated p21 and CCND1 mRNA and protein levels in the different MSC cell lines tested (Figure 6D). However, the E2F1 mRNA levels were all significantly low in all the three cell lines tested (Figure 6D, left panel); however, the E2F1 protein level was lower than the control only in WJ2000 cells, but no significant changes were observed in the other two cell lines on H_2_O_2_ treatment (Figure 6D, right panel). Taken together, the data show that miR-20b-5p and miR-106a-5p target and down-regulate expression of p21, CCND1 and E2F1, and that oxidative stress exerts the reverse effects in up-regulating p21 and CCND1 expression, but not E2F1. Both the miRNA and oxidative stress effects may be linked to their effects on suppressed cell proliferation and DNA synthesis and hampered G1/S transition described above.

## Discussion

### Oxidative stress-deregulated miR-17 family miRNAs are frequently associated with cancers and senescence

In this study, twelve miRNAs that are significantly deregulated in oxidative stress-induced senescence cells were identified (Table 1), many of which are predicted to target genes associated with various cancers (Figure 1B; Supplementary Table S3). MiR-146a has previously been shown to be up-regulated by p53-binding protein-1 to regulate NF-kB activities associated with metastasis of xenografted breast cancer cells in nude mice 48. Besides the two miR-146 family miRNAs, the other five up-regulated miRNAs were first reported in deep sequencing of small RNA transcriptomes of malignant B cells, melanoma and breast cancers 32-36, and have not been fully annotated and investigated. It is, however, noteworthy that the gene encoding miR-4732-5p is located in close proximity to the *ERBB2* receptor gene, which is frequently amplified and over-expressed in breast cancer cells 34; miR-4732-5p may be collaterally amplified in cancer cells. In the down-regulated miRNA group (Table 1), miR-16 is a well-characterized tumor suppressor that regulates the cell cycle and apoptosis 37. On the other hand, the miR-17 family miRNAs are deregulated in a number of cancers, and have been implicated as oncomirs 38,49. The four miR-17 family members are paralogous genes located in the miR-17~92 and miR-106a~363 miRNA clusters on chromosomes 13 and X, respectively 38, and have been extensively studied in their roles in tumorigenesis 49-53. Deregulation of the validated miR-17~92 targets, including E2F1, TP53INP1, TGFβ and PTEN, is known to contribute to uncontrolled cell proliferation, tumor growth, apoptosis and metastasis 39,49. Taken together, various reports, including this study, on the deregulated expression of the miR-17 family miRNAs are consistent with the notion that oxidative stress, and possibly cellular senescence, share some common features with the tumorigenesis process 3-4, 54,55.

In the up-regulated miRNA group, deregulated expression of miR-146a has previously been shown in long-term culturing or late-passage replicative senescence human fibroblast and endothelial cells 31,56. Besides possible roles in tumorigenesis, the chromosome 13-associated miR-17 and miR-20a have also previously been investigated in replicative aging models, including MSC 57. The chromosome X-associated miR-20b-5p and miR-106a-5p were shown in this work to target both pro- and anti-proliferative proteins (Figure 4), echoing previous reports 30,38-40. In stress-induced premature senescence fibroblasts, miR-17 suppresses p21 expression 58, as was also observed for miR-20b-5p and miR-106a-5p, in this work (Figure 6C).

### A proposed scheme on miR-20b-5p/miR-106a-5p-dependent and -independent regulation of E2F1 expression and G1/S transition under oxidative stress

To investigate the role of miRNA in oxidative-stressed cells in this work, miR-20a-5p and miR-106a-5p were first identified to be down-regulated in H_2_O_2_-treated cells (Figures 1 & 2). H_2_O_2_ treatment was further shown to suppress G1/S transition and DNA synthesis (Figure 3) whereas the opposite effects were shown when miR-20b-5p and miR-106a-5p were over-expressed (Figure 4). Since miRNAs are negative regulators, the H_2_O_2_ and miRNA data are consistent. MiR-20a-5p and miR-106a-5p targeting of p21, CCND1 and E2F1 was predicted by bioinformatics analysis (Figure 5), and was confirmed in luciferase assays (Figures 6A & 6B) and in correlation with down-regulation of the three targets on miRNA over-expression (Figure 6C). On H_2_O_2_ treatment, p21 and CCND1 levels were up-regulated (Figure 6D). However, H_2_O_2_ treatment resulted in suppressed E2F1 mRNA levels in all the three MSC cell lines tested, and suppressed E2F1 protein level only in WJ2000 cells, but the E2F1 levels were not significantly altered in the other two cell lines (Figure 6D).

**Figure 7:**
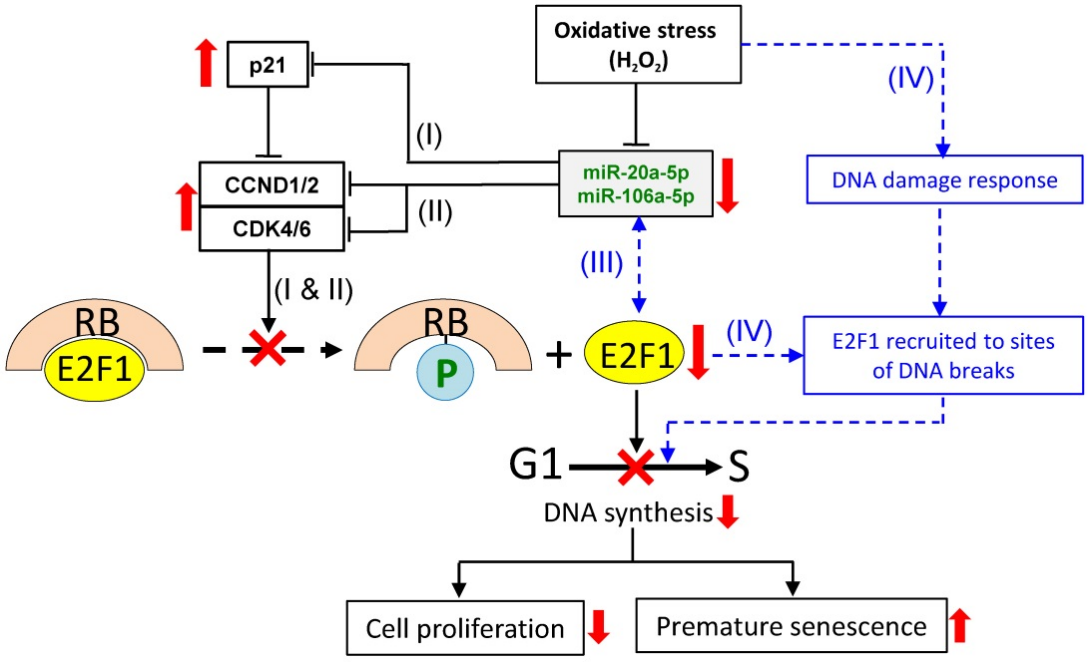
A proposed scheme on miR-20b-5p/miR-106a-5p-dependent and -independent regulation of E2F1 cellular levels and G1/S transition under oxidative stress. To regulate the E2F1 level under oxidative stress, three miRNA-dependent (I, II and III) and a miRNA-independent (IV) routes are proposed. Routes (I) & (II): the down-regulated miRNAs lead to up-regulated expression of p21 (I) and CCND1/2 and CDK4/6 (II) independently; route (III): miRNA-E2F1 auto-regulatory feedback regulation; route (IV): oxidative stress induces DNA damage response leading to recruitment of E2F1 to DNA break sites. The combined effects of the four pathways result in E2F1 down-regulation, inhibition of G1/S transition and DNA synthesis, suppressed cell proliferation and premature senescence. See text for further description of the proposed scheme. Solid lines indicate events that are supported by evidences presented in this work, as are up- and downward-pointing red arrows that indicate up- or down-regulated gene expression, and the red crosses indicate suppression of the indicated cellular processes. Blue dashed lines and lettering indicate proposed regulatory events that have been described in the literature.

Based on the data presented in this and a previous work 12, and also drawing literature on the analysis of other miR-17 members, a scheme is proposed here to explain the E2F1 response and in relation to miR-20a-5p/miR-106a-5p expression under oxidative stress. It is proposed that under oxidative stress, four separate but inter-related miR-20a-5p/miR-106a-5p-dependent and -independent events collectively modulate E2F1 expression and G1/S transition to affect cell proliferation and oxidative stressed-induced premature senescence (Figure 7). In event (I), oxidative stress down-regulates expression of miR-20b-5p and miR-106a-5p, which, in turn, up-regulates p21 expression. In event (II), the two miRNAs independently and individually target and regulate CCND1/2 and CDK4/6. Despite the fact that p21 is an inhibitor of CCND1/2-CDK4/6, CCND1/2 is still up-regulated, and the E2F1 levels are suppressed. To explain the discrepancies, event (III) is proposed based on reports of an auto-regulatory feedback loop between E2F factors and other miR-17 family members, viz. miR-17-5p and miR-20a, and also other miRNAs 59-62. MiR-20b-5p/miR-106a-5p, being members of the same miR-17 family, and E2F1 may also be under auto-regulatory feedback control under oxidative stress in that the miRNAs inhibit E2F1 expression, and, on the other hand, E2F1 binds to the promoter of the miR-17 miRNA clusters to block miRNA expression. Event (IV) is miRNA-independent in which oxidative stress triggers DNA damages, which activate the DNA damage response to recruit E2F1 and CCND1 to the sites of the DNA breaks to promote DNA repair 63-64, and to initiate temporary cell cycle arrest under oxidative stress 65-66. Hence, the E2F1 levels in the H_2_O_2_-treated cells are proposed to be the outcome of the cumulative effects of the interactions and balances between the four miRNA-dependent and -independent events, and are subjected to further modulation by multiple factors, including the physiological state of the cell and the relative spatial- and temporal-dependent abundance of specific factors. Besides affecting the G1/S transition of the cell cycle reported here, H_2_O_2_-induced oxidative stress may also exert influences on MSC differentiation (reviewed in 67) and other cellular process via miRNA regulation. These are important issues worthy of further investigations.

## Conclusions

Global miRNA profiling of MSC under oxidative stress and KEGG pathway analysis in this work lend further support that oxidative stress share numerous affected target genes with the tumorigenesis process. This work also shows that oxidative stress down-regulates expression of the miR-17 family miRNAs, and that the miR-17 family members, miR-20b-5p and miR-106a-5p, inhibit expression of E2F1 via targeting p21 and CCND1 to suppress the G1/S-phase transition of the cell cycle, possibly resulting in premature senescence. Understanding the role of the miR-17 family miRNAs under oxidative stress may lead to the development of strategies to retard or reverse senescence in MSC culture, and to facilitate regenerative medicine.

## Supplementary Material

Supplementary tables.

## Figures and Tables

**Table 1:** Deregulated miRNAs in H_2_O_2_-induced oxidative- stressed multipotent stromal cells

miRNA	Family	Accession no	Chromosomal location	Log_2_(FC)
**Up-regulated miRNA (n=7)**				
miR-146a-5p	miR-146	MI0000477	5q34	2.407±1.51*
miR-146b-5p	miR-146	MI0003129	10q24.32	2.029±1.44*
miR-1587	NA	MI0016905	Xp11.4	1.019±0.15**
miR-3178	NA	MI0014212	16p13.3	1.043±0.39**
miR-4497	NA	MI0016859	12q24.1	1.179±0.36**
miR-4508	NA	MI0016872	15q11.2	1.055±0.06**
miR-4732-5p	NA	MI0017369	17q11.2	1.138±0.77*
**Down-regulated miRNA (n=5**)				
miR-16-5p	miR-15	MI0000070	13q14.2	-1.281±0.85*
miR-17-5p	miR-17	MI0000071	13q31.3	-1.268±0.82*
miR-20a-5p	miR-17	MI0000076	13q31.3	-1.265±0.81**
miR-20b-5p	miR-17	MI0001519	Xq26.2	-1.217±0.76**
miR-106a-5p	miR-17	MI0000113	Xq26.2	-1.158±0.70**

Microarray data of log_2_(fold change) (FC) > 1.00 and < -1.00, and *p < 0.05 or **p < 0.01, were extracted from Supplementary Table S2. The miRNAs shown were differentially expressed in all the three cell lines analyzed. NA, not assigned.

**Table 2:** Predicted regulatory processes and gene counts targeted by the deregulated miRNAs in oxidative-stressed MSC

Regulatory process	miRNA expression	Gene count
**Gene regulation**		
GO:0000978~RNA pol II core promoter proximal region sequence-specific DNA binding	Up	23
GO:0001078~Transcriptional repressor activity, RNA pol II core promoter proximal region sequence -specific binding	Up	6
GO:0003676~Nucleic acid binding	Up	38
GO:0000289~Nuclear-transcribed mRNA poly(A) tail shortening	Down	4
GO:0045787~Positive regulation of cell cycle	Down	5
GO:0035194~Posttranscriptional gene silencing by RNA	Down	4
GO:0035278~miRNA mediated inhibition of translation	Down	5
	Total	85 (32.9%)
**Kinase activity and signaling**		
GO:0008589~Regulation of smoothened signaling pathway	Up	4
GO:0038083~Peptidyl-tyrosine autophosphorylation	Up	6
GO:0045747~Positive regulation of Notch signaling pathway	UP	4
GO:0004674~Protein serine/threonine kinase activity	Down	20
GO:0004709~MAP kinase kinase kinase activity	Down	4
GO:0004715~Non-membrane spanning protein tyrosine kinase activity	Down	4
	Total	42 (16.3%)
**Cell cycle**		
GO:0045787~Positive regulation of cell cycle	Down	5
	Total	5 (1.9%)

Data is extracted from the biological and molecular processes in the gene ontology analysis of the significantly and differentially expressed miRNAs in OSIPS cells as presented in Supplementary Table S3. Combining the up- and down-regulated biological and molecular processes, there are 258 genes; the percentages shown in brackets are percentages of the total gene counts.

## Abbreviations

CCND1/2: cyclin D1/D2
CDK: cyclin-dependent kinase
E2F: E2F transcription factor
GAPDH: glyceraldehyde-3-phosphate dehydrogenase
H_2_O_2_: hydrogen peroxide
miRNA: microRNA
MSC: multipotent stromal cells
NF-kB: nuclear factor kappa-light-chain-enhancer of activated B cells
OSIPS: oxidative stress-induced premature senescence
p21: cyclin-dependent kinase inhibitor 1A
PTEN: phosphatase and tensin homolog
RB1: retinoblastoma protein 1
TGFβ: transforming growth factor, beta
WJ-MSC: Wharton's Jelly-derived MSC
