# Supplementary material for: Oxidative Stress Down-Regulates MiR-20b-5p, MiR-106a-5p and E2F1 Expression to Suppress the G1/S Transition of the Cell Cycle in Multipotent Stromal Cells
Source: Int J Med Sci. 2020 Feb 4;17(4):457–70. doi: 10.7150/ijms.38832 (PMC7053300; doi:10.7150/ijms.38832)
Supplement: Supplementary file 1 — Supplementary tables. [file ijmsv17p0457s1.pdf]

Supplementary Table S1. Sequences of miRNA primers used in the study

**miRNA**	**Primer sequences (5'-3')**
miR-17-5p	CAAAGTGCTTACAGTGCAGGTAG
miR-20a-5p	TAAAGTGCTTATAGTGCAGGTAG
miR-20b-5p	CAAAGTGCTCATAGTGCAGGTAG
miR-106a-5p	AAAAGTGCTTACAGTGCAGGTAG
U6	CACCACGTTTATACGCCGGTG

miRNA sequences were derived from miRBase version 20.

Supplementary Table S2. Deregulated miRNAs in H_2O_2-induced oxidative-stressed MSC

miRNA	Family	Acc. No	Chromosomal location	Sequence (5' → 3')	Log_2(FC)
**Up-regulated miRNA (n=28)**					
miR-146a-5p	miR-146	MI0000477	5q34	ugagaacugaauuccaugggguu	2.407*
miR-146b-5p	miR-146	MI0003129	10q24.32	ugagaacugaauuccauaggcu	2.029*
miR-4497	NA	MI0016859	12q24.1	cuccgggacggcugggc	1.179**
miR-513a-5p	miR-506	MI0003191	Xq27.3	uucacagggaggugucau	1.142
miR-4732-5p	NA	MI0017369	17q11.2	uguagagcagggagcaggaagcu	1.138*
miR-4508	NA	MI0016872	15q11.2	gcggggcugggcgcgcg	1.055**
miR-3178	NA	MI0014212	16p13.3	ggggcgcggccggaucg	1.043**
miR-1587	NA	MI0016905	Xp11.4	uugggcugggcugguuggg	1.019**
miR-4475	NA	MI0016827	9	caagggaccaagcauucauuau	0.934*
miR-513b	miR-506	MI0006648	Xq27.3	uucacaaggaggugucauuuau	0.905
miR-4299	NA	MI0015829	11	gcuggugacaugagaggc	0.817*
miR-921	miR-921	MI0005713	1q24.1	cuagugagggacagaaccaggauuc	0.792
miR-433	miR-433	MI0001723	14q32.2	uacggugagccugucauuauuc	0.790
miR-3621	NA	MI0016012	9	cgcgggucggggucugcagg	0.773
miR-5191	NA	MI0018170	1	aggauaggaagaaugaagugcu	0.745
miR-34a-5p	miR-34	MI0000268	1p36.22	uggcagugucuuagcugguugu	0.733
miR-4484	miR-4484	MI0016845	10	aaaaggcgggagaagcccca	0.623
miR-4511	NA	MI0016877	15	gaagaacuguugcauuugcccu	0.607
miR-4788	miR-4788	MI0017435	3	uuacggaccagcuaagggaggc	0.441
miR-221-3p	miR-221	MI0000298	Xp11.3	agcuacauugucugcuggguuuc	0.393
miR-4449	miR-4449	MI0016792	4	cgucccggggcugcgcgaggca	0.373
miR-4288	NA	MI0015896	8	uugucugcugaguuucc	0.366
miR-1275	miR-1275	MI0006415	6	gugggggagaggcuguc	0.294
miR-4795-5p	NA	MI0017442	3	agaaguggcuaauaauauuga	0.246
miR-4423-3p	miR-4423	MI0016760	1	auaggcaccaaaaagcaacaa	0.187
miR-1973	NA	MI0009983	4	accgugcaaagguagcaua	0.084
miR-29a-3p	miR-29	MI000087	7q32.3	uagcaccaucugaaaucgguua	0.060
miR-181a-2-3p	miR-181	MI0000269	9q33.3	accacugaccguugacuguacc	0.018

Down-regulated miRNA (n=22)					
miR-16-5p	miR-15	MI0000070	13q14.2	uagcagcacguaaauauuggcg	-1.281*
miR-17-5p	miR-17	MI0000071	13q31.3	caaagugcuuacagugcagguag	-1.268*
miR-20a-5p	miR-17	MI0000076	13q31.3	uaaagugcuuauagugcagguag	-1.265**
miR-20b-5p	miR-17	MI0001519	Xq26.2	caaagugcucauagugcagguag	-1.217**
miR-106a-5p	miR-17	MI0000113	Xq26.2	aaaagugcuuacagugcagguag	-1.158**
miR-106b-5p	miR-17	MI0000734	7q22.1	uaaagugcugacagugcagau	-0.943**
miR-19b-3p	miR-19	MI0000074	13q31.3	ugugcaaauccaugcaaaacuga	-0.927**
miR-15b-5p	miR-15	MI0000438	3q35.33	uagcagcacaucaugguuuaca	-0.883*
miR-663a	miR-663	MI0003672	20p11.1	aggcggggcgccgcgggaccgc	-0.827
miR-665	miR-665	MI0005563	14q32.2	accaggaggcugaggccccu	-0.766
miR-4521	NA	MI006887	17	gcuaaggaaguccugugcucag	-0.697
miR-3687	miR-3687	MI0016088	21	cccggacaggcguucgugcgacgu	-0.685
miR-3158-5p	miR-3158	MI0014186	10	ccugcagagaggaagcccuuc	-0.582
miR-424-5p	miR-322	MI0001446	Xq26.3	cagcagcaauucauguuuugaa	-0.530
miR-29b-3p	miR-29	MI0000105	7q32.3	uagcaccauuugaaaucaguguu	-0.515
miR-186-3p	miR-186	MI0000483	1p31.1	gcccaaaggugaauuuuuuggg	-0.404
miR-4271	NA	MI0015879	3	gggggaagaaaaggugggg	-0.384
miR-483-3p	miR-483	MI0002467	11p15.5	ucacuccucuccucccgucuu	-0.270
miR-31-3p	miR-31	MI0000089	9p21.3	ugcuaugccaacauauugccau	-0.214
miR-193a-3p	miR-193	MI0000487	17q11.2	aacuggccuacaaagucccagu	-0.155
miR-548a-3p	miR-548	MI0019132	13	uaaaacccacaauuauguuugu	-0.146
miR-4635	NA	MI0017262	5	ucuugaagucagaacccgcaa	-0.098

miRBase version 20 was used in data derivation. Included are microarray data of $\log_2$(fold change, FC) between <-1 and >1.00, and *P<0.05, **P<0.01. NA, not assigned.

Supplementary Table S3. Gene ontology and KEGG pathway analysis of the deregulated miRNAs in **H_2O_2-induced oxidative-stressed MSC**

A. Down-regulated miRNAs

Biological Process

Process	No. of genes
GO:0035194~Posttranscriptional gene silencing by RNA	4
GO:0060078~Regulation of postsynaptic membrane potential	4
GO:0045737~Positive regulation of cyclin-dependent protein serine/threonine kinase activity	4
GO:0070372~Regulation of ERK1 and ERK2 cascade	4
GO:0019228~Neuronal action potential	4
GO:2000145~Regulation of cell motility	4
GO:0003281~Ventricular septum development	4
GO:0000289~Nuclear-transcribed mRNA poly(A) tail shortening	4
GO:0035278~miRNA mediated inhibition of translation	5
GO:0045787~Positive regulation of cell cycle	5
Total	42

Molecular Function

Function	No. of genes
GO:0005248~Voltage-gated sodium channel activity	4
GO:0004709~MAP kinase kinase kinase activity	4
GO:0003785~Actin monomer binding	4
GO:0004715~Non-membrane spanning protein tyrosine kinase activity	4
GO:0043022~Ribosome binding	4
GO:0005089~Rho guanyl-nucleotide exchange factor activity	5
GO:0004843~Thiol-dependent ubiquitin-specific protease activity	5
GO:0004725~Protein tyrosine phosphatase activity	6
GO:0001078~Transcriptional repressor activity, RNA polymerase II core promoter proximal region sequence-specific binding	6
GO:0004674~Protein serine/threonine kinase activity	20
Total	62

KEGG Pathway

Pathway	No. of genes
hsa05223~Non-small cell lung cancer	5
hsa05211~Renal cell carcinoma	5
hsa05219~Bladder cancer	6
hsa05212~Pancreatic cancer	6
hsa05214 ~Glioma	6
hsa05218~Melanoma	6
hsa05220~Chronic myeloid leukemia	7
hsa05215~Prostate cancer	7
hsa04012~ErbB signaling pathway	8
hsa04110~Cell cycle	9
Total	65

B. Up-regulated miRNAs

Biological Process

Process	No. of genes
GO:0036120~Cellular response to platelet-derived growth factor stimulus	4
GO:0008589~Regulation of smoothened signaling pathway	4
GO:0017157~Regulation of exocytosis	4
GO:0045747~Positive regulation of Notch signaling pathway	4
GO:0048791~Calcium ion-regulated exocytosis of neurotransmitter	4
GO:0031397~Negative regulation of protein ubiquitination	4
GO:0071277~Cellular response to calcium ion	5
GO:0038083~Peptidyl-tyrosine autophosphorylation	6
GO:0071230~Cellular response to amino acid stimulus	6
GO:0042733~Embryonic digit morphogenesis	6
Total	47

Molecular Function

Function	No. of genes
GO:0005112~Notch binding	4
GO:0005154~Eepidermal growth factor receptor binding	5
GO:0004715~Non-membrane spanning protein tyrosine kinase activity	5
GO:0030145~Manganese ion binding	5
GO:0008237~Metallopeptidase activity	5

GO:0001078~Transcriptional repressor activity, RNA polymerase II core promoter proximal region sequence-specific binding	6
GO:0004222~Metalloendopeptidase activity	7
GO:0005516~Calmodulin binding	9
GO:0000978~RNA polymerase II core promoter proximal region sequence-specific DNA binding	23
GO:0003676~Nucleic acid binding	38
Total	107

KEGG Pathway

Pathway	No. of genes
hsa05217 ~Basal cell carcinoma	4
hsa05416~Viral myocarditis	4
hsa05210~Colorectal cancer	4
hsa04918 ~Thyroid hormone synthesis	5
hsa04520 ~Adherens junction	5
hsa04012~ErbB signaling pathway	5
hsa04070~Phosphatidylinositol signaling system	5
hsa05205 ~Proteoglycans in cancer	8
hsa04810 ~Regulation of actin cytoskeleton	9
hsa05200 ~Pathways in cancer	12
Total	61

Online databases, including TargetScan v7.1, microT-CDS v5.0 and miRTarBase, were used to predict the genes that are targeted by the seven up-regulated and five down-regulated miRNAs (Table 1). A total of 437 genes in the up-regulated group and 405 genes in the down-regulated group were selected for gene ontology (GO) and KEGG pathway analysis by using DAVID Bioinformatics Resources v6.8. For the generation of the functional annotation chart, the threshold of minimum gene counts belonging to an annotation term was set at ≥ 4 and the Ease score threshold (modified Fisher Exact P-value) was set at 0.1. Top ten biological process, molecular function and KEGG terms with the highest fold enrichment score were then selected and tabulated.
